# Inhibition of Intracellular ROS Accumulation by Formononetin Attenuates Cisplatin-Mediated Apoptosis in LLC-PK1 Cells

**DOI:** 10.3390/ijms19030813

**Published:** 2018-03-12

**Authors:** Haesol Lee, Dahae Lee, Ki Sung Kang, Ji Hoon Song, You-Kyoung Choi

**Affiliations:** 1 Department of Korean International Medicine, College of Korean Medicine, Gachon University, Seongnam 13120, Korea; haesol89@naver.com; 2 College of Korean Medicine, Gachon University, Seongnam 13120, Korea; pjsldh@naver.com (D.L.); kkang@gachon.ac.kr (K.S.K.); 3 Department of Medicine, University of Ulsan College of Medicine, Seoul 05505, Korea

**Keywords:** formononetin, nephrotoxicity, cisplatin, apoptosis, LLC-PK1 cell, cervical cancer cells

## Abstract

Cisplatin is a well-known anticancer drug frequently used for treating solid tumors, including ovarian, testicular, bladder, and cervical tumors. However, usage of cisplatin has been limited because of its adverse effects, particularly nephrotoxicity. Therefore, the present study sought to investigate the protective effect of formononetin against cisplatin-induced cytotoxicity in LLC-PK1 pig kidney epithelial cells as well as the anticancer effect of cisplatin in three different human cervical cancer cell lines, including HeLa, SiHa, and CaSKi cells. We first demonstrated that formononetin strongly prevented cisplatin-induced LLC-PK1 cell death. Although formononetin had no anticancer effect, it did not interrupt the anticancer effect of cisplatin in human cervical carcinoma cell lines. Furthermore, the treatment with formononetin reduced reactive oxygen species (ROS) accumulation and chromatin condensation. The percentage of Annexin V-positive cells also increased following cisplatin treatment. Finally, formononetin-inhibited c-Jun N-terminal kinase (JNK) phosphorylation, cleavage of caspase-8 and caspase-3, and the ratio of Bax to Bcl-2 increased with cisplatin. Taken together, these findings suggest that formononetin may be a possible option to prevent nephrotoxicity induced by cisplatin during treatment for cervical cancer.

## 1. Introduction

Cisplatin is the most effective chemotherapeutic agent for various solid cancers, including testicular cancer, ovarian cancer, lung cancer, prostate cancer, breast cancer, and head and neck cancer [1,2,3]. Although cisplatin has strong anticancer effects, its usage has been limited as a result of its severe side effects. Nephrotoxicity is a major adverse effect of cisplatin treatment for cancers [4]. Cisplatin mainly induces epithelial cell damage in the kidney [5]. Cisplatin-mediated nephropathy is associated with various risk factors, including dosage and frequency of cisplatin administration. At higher doses, cisplatin may contribute to the reduction of glomerular filtration rate and plasma magnesium concentration [6,7].

Evidence increasingly suggests that cisplatin-induced nephrotoxicity occurs through multiple molecular pathways. Among these, oxidative stress is implicated in cisplatin-induced cell damage in the kidney. Cisplatin treatment interrupts the balance between oxidants and antioxidants, resulting in oxidative stress in the kidney. In contrast, cisplatin-mediated oxidative stress is prevented by antioxidants, such as selenium, flavonoids, vitamins, and *N*-acetylcysteine (NAC) [8,9,10]. Cisplatin-induced oxidative stress also contributes to apoptosis and necrosis at lower and higher concentrations of cisplatin, respectively [11,12]. Caspases play a pivotal role in cisplatin-induced apoptosis in renal tubular epithelial cells. Previous reports have demonstrated that cisplatin activates caspase-8 and caspase-3, and that inhibition of caspases by generic and pharmacological inhibitors prevents cisplatin-induced cell death [13,14]. Furthermore, cisplatin-induced cell death is reportedly associated with an increase in the ratio of Bax to Bcl-2 protein expression [15]. Activation of mitogen-activated protein kinases (MAPKs), including extracellular signal-regulated kinase (ERK), c-Jun N-terminal kinase (JNK), and p38 is also involved in cisplatin-induced apoptosis [16].

**Figure 1:**
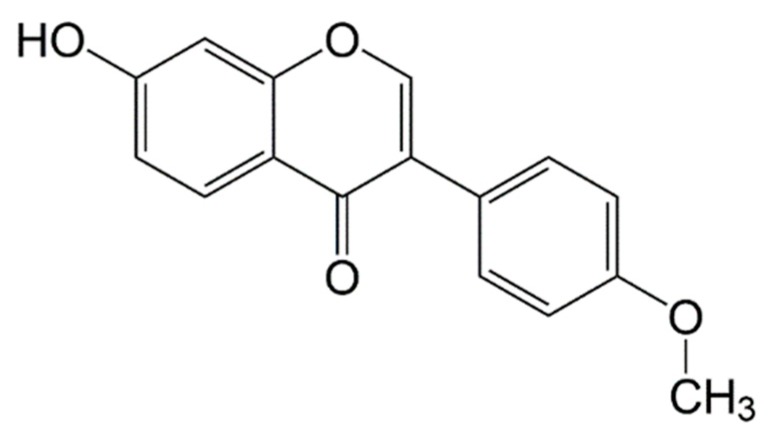
Chemical structure of formononetin (FOR).

Formononetin, an *O*-methylated isoflavone, is one of the major bioactive compounds in red clover plants (Figure 1). Although recent reports suggest that formononetin prevents acute cisplatin-induced renal injury [17], the underlying molecular mechanism has not been clearly demonstrated. Thus, this study was conducted to assess the effect of formononetin and to demonstrate its protective mechanism against cisplatin-induced LLC-PK1 pig kidney epithelial cell death.

## 2. Results and Discussion

### 2.1. The Effect of Formononetin in Cisplatin-Induced Cell Death in LLC-PK1 Cells

**Figure 2:**
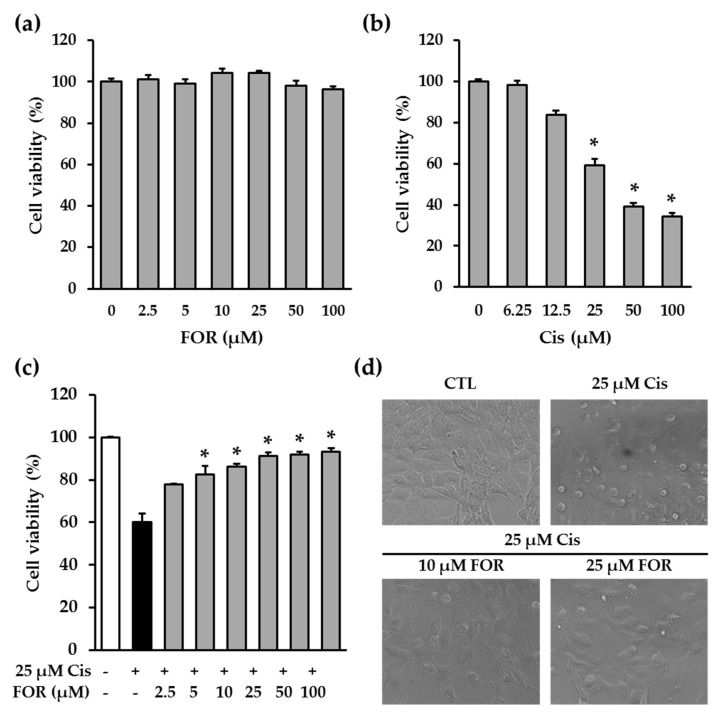
Formononetin prevents cisplatin-induced LLC-PK1 cell death. (**a**) LLC-PK1 cells were treated with the indicated concentrations of formononetin and cell viability was determined. Bars denote the percentage of cell viability (mean ± S.E.M.). (**b**) LLC-PK1 cells were treated with the indicated concentrations of cisplatin (Cis) and cell viability was determined. Bars denote the percentage of cell viability (mean ± S.E.M., * *p* < 0.05 compared with cisplatin-treated cells). (**c**) LLC-PK1 cells were exposed to 25 μM cisplatin for 24 h in the presence of indicated concentrations of formononetin and cell viability was determined. Bars denote the percentage of cell viability (mean ± S.E.M., * *p* < 0.05 compared with cisplatin-treated cells). (**d**) Morphological changes were observed under the microscope.

Although cisplatin is one of the most effective chemotherapeutic agents to treat various solid tumors, side effects include the inducement of nephrotoxicity. Therefore, the prevention of nephrotoxicity may be beneficial to ameliorate the defect of cisplatin. This study initially elucidated the protective effect of formononetin on cisplatin-induced nephrotoxicity using LLC-PK1 renal tubular epithelial cells. Our result indicated that formononetin was not cytotoxic in LLC-PK1 cells (Figure 2a) and that cisplatin diminished the cell viability of LLC-PK1 cells in a concentration-dependent manner (Figure 2b). In addition, our previous report optimized the concentration of cisplatin that causes apoptosis in LLC-PK1 cells [18]. Thus, to examine whether formononetin prevents cisplatin-induced cytotoxicity under these conditions, LLC-PK1 cells were treated with 25 μM cisplatin in the presence of indicated concentrations of formononetin and a cell viability assay was conducted. Treatment with cisplatin reduced cell viability to 60.28%, whereas the presence of formononetin showed a significant increase in the viability of LLC-PK1 cells. The strongest protective effect of formononetin was demonstrated from 25 µM to 100 µM (89.25 ± 2.12%, 91.85 ± 1.48%, and 93.25 ± 1.49%, respectively) (Figure 2c). However, by itself, formononetin had no effect on cell viability in LLC-PK1 cells (Figure 2b). In addition, formononetin-treated cells were morphologically normal compared with cells undergoing cisplatin-induced death (Figure 2d). These results indicate that formononetin is a potent protective agent against cisplatin-mediated LLC-PK1 cell death.

### 2.2. Formononetin Did Not Alter the Anticancer Effect of Cisplatin on Three Different Cervical Cancer Cell Lines, Including HeLa, SiHa, and CaSKi 

**Figure 3:**
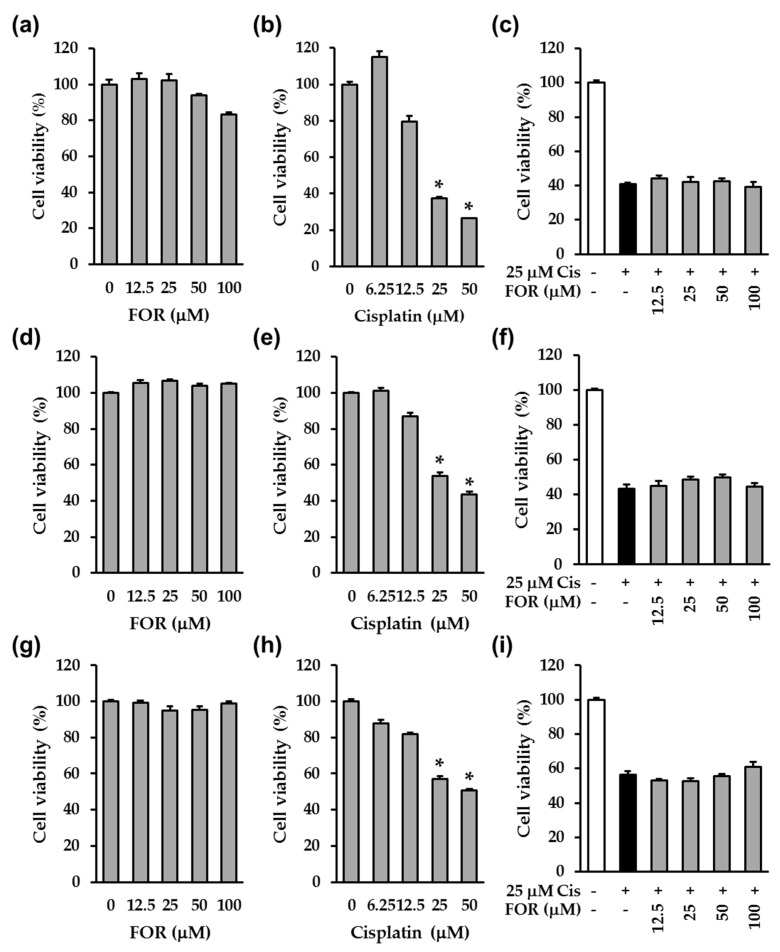
Formononetin had no effect on the anticancer effect of cisplatin in cervical cancer cells. (**a**–**c**) HeLa cells were exposed to formononetin (**a**), cisplatin (**b**), or formononetin with 25 μM cisplatin (**c**) for 24 h. (**d**,**f**) SaHa cells were exposed to formononetin (**d**), cisplatin (**e**), or formononetin with 25 μM cisplatin (**f**) for 24 h. (**g**–**i**) CaSKi cells were exposed to formononetin (**g**), cisplatin (**h**), or formononetin with 25 μM cisplatin (**i**) for 24 h. Bars denote the percentage of cell viability (mean ± S.E.M., * *p* < 0.05 compared with control cells).

In addition, we examined the anticancer effect of cisplatin, formonetin, or cisplatin combined with formononetin on three different cervical cancer cell lines, such as HeLa, SiHa, and CaSKi cells. Our results showed that the treatment with cisplatin significantly reduced the cell viability while fornononetin did not affect the HeLa, SiHa, and CaSKi cells (Figure 3a–f). Furthermore, formononetin did not affect the anticancer effect of cisplatin in HeLa, SiHa, and CaSKi cells (Figure 3g–i). These results indicate that formononetin retained the anticancer effect of cisplatin and effectively prevented cisplatin-induced LLC-PK1 cell death. Therefore, we mainly focused on demonstrating the protective mechanism of formononetin against cisplatin-induced LLCP-K1 cell death.

### 2.3. Formononetin Prevented Intracellular Reactive Oxygen Speiceses (ROS) Accumulation

**Figure 4:**
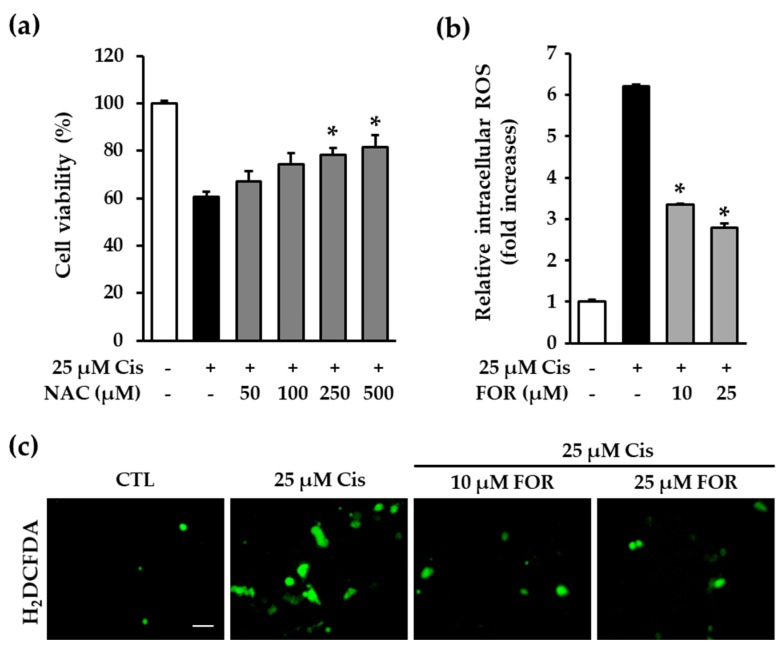
Formononetin inhibits cisplatin-induced oxidative stress. (**a**) LLC-PK1 cells were treated with 25 μM cisplatin in the presence or absence of the NAC and carried out cell viability assay. Bars denotes the percentage of cell viability (mean ± S.E.M., * *p* < 0.05 compared with cisplatin-treated cells); (**b**) LLC-PK1 cells exposed to 25 μM cisplatin for 24 h with formononetin followed by staining cells with H_2_DCFDA. Bar graphs depict the fold increases in the intracellular ROS (mean ± S.E.M., * *p* < 0.05 compared with cisplatin-treated cells); (**c**) fluorescence microscopic images indicated the intracellular ROS accumulation. Green color indicates DCF-positive cells. Scal bar, 50 µm.

Reports suggest that cisplatin triggers excessive intracellular ROS accumulation resulting in tubular epithelial cell death in the kidney as well as that inhibition of oxidative stress prevents cisplatin-induced nephrotoxicity [19,20]. A previous report demonstrated that *N*-acetyl-cysteine (NAC), a well-known antioxidant, prevented cisplatin-induced toxicity in an animal model [8]. Therefore, we examined whether formononetin could prevent ROS accumulation triggered by cisplatin. Consistent with previous reports, our results showed that NAC prevented the decrease in cell viability induced by cisplatin (Figure 4a). These data suggest that formononetin-mediated protection against LLC-PK1 cell death may be associated with the prevention of oxidative stress.

Oxidative stress is one of the multiple pathways involved in cisplatin-induced nephropathy and is ameliorated by antioxidants such as NAC [8,12]. Therefore, we examined whether formononetin exerts an antioxidative activity against cisplatin-induced oxidative stress. LLC-PK1 cells were exposed to cisplatin along with 10 μM and 25 μM formononetin for 24 h and then stained with 2′,7′-dichlorofluorescin diacetate (H_2_DCFDA), a membrane-permeable indicator for ROS. Quantitatively analyzed data showed that the treatment with 10 or 25 μM formononetin significantly reduced the fluorescence intensity of DCF enhanced by cisplatin (Figure 4b). The representative fluorescence images revealed that DCF-positive cells were increased markedly by cisplatin treatment, whereas the presence of formononetin prevented this effect (Figure 4c). A recent study reported that formononetin enhanced epirubicin-induced HeLa cell death by increasing ROS formation [21]. However, another paper indicated that the blockade of intracellular ROS formation by formononetin prevented oxygen-glucose deprivation and reoxygenation-induced rat cardiomyocyte H9c2 cell death [22]. Based on these studies, formononetin functions differently depending on experimental conditions. Therefore, our findings suggest that formononetin exerts strong antioxidative activity against cisplatin-induced oxidative stress in at least LLC-PK1 cells.

### 2.4. Formononetin Prevented Cisplatin-Induced Apoptotic LLC-PK1 Cell Death

**Figure 5:**
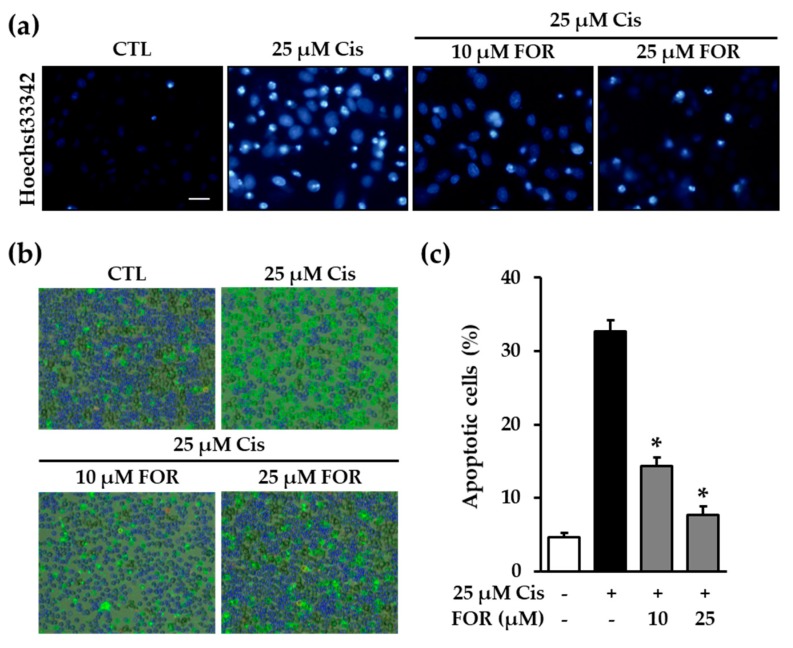
Formononetin attenuates cisplatin-induced apoptosis in LLC-PK1 cells. (**a**) LLC-PK1 cells were exposed to 25 μM cisplatin for 24 h in the presence of formononetin followed by staining with Hoechst-33342. Bright blue indicates the chromatin condensation. Scale bar, 50 μm; (**b**) representative images (40× magnification) were obtained using Tali-image–based cytometric analysis to determine the apoptotic cells (blue: live cells, green color: Annexin V positive cells); (**c**) bars indicate the percentage of apoptotic cells (mean ± S.E.M., * *p* < 0.05 compared with cisplatin-treated cells).

Another pathway involved in cisplatin-mediated nephrotoxicity is the apoptotic pathway. Apoptosis is defined as programmed cell death and is morphologically characterized by cell shrinkage, chromatin condensation, and membrane budding [23,24]. Based on our recent studies, inhibition of apoptotic cell death can be a target for renoprotection [18,25,26]. Therefore, this study was conducted to demonstrate the effect of formononetin against cisplatin-induced apoptosis. Cells were stained with Hoechst-33342 to detect chromatin condensation after exposure to cisplatin for 24 h in the presence of 10 μM and 25 μM formononetin. As shown in Figure 3a, the exposure to cisplatin dramatically increased chromatin condensation in LLC-PK1 cells, whereas formononetin treatment markedly inhibited chromatin condensation (Figure 5a). Cells were also stained with Annexin V Alexa Fluor 488 and propidium iodide to analyze the proportion of apoptotic cells using image-based cytometric analysis. Annexin V binds to phosphatidylserine which is exposed on the outer membrane of apoptotic cells [27]. Annexin V-positivity indicates the presence of apoptotic cells. Our results showed that the number of Annexin V-positive cells was markedly increased after exposure to cisplatin for 24 h, whereas it was reduced in the presence of formononetin (Figure 5b). Moreover, quantitative analysis of the data revealed that the proportion of apoptotic cells was significantly diminished in the presence of 10 μM and 25 μM formononetin (14.3 ± 1.1% and 7.6 ± 1.1%, respectively) compared to cisplatin exposure alone (Figure 5c). These findings indicate that formononetin effectively attenuates cisplatin-induced apoptosis in LLC-PK1 cells.

### 2.5. Formononetin Inhibited Cisplatin-Meidiated Phosphorylation of JNK, Cleavage of Caspase-8 and Caspase-3, and the Ratio of Bax to Bcl-2

**Figure 6:**
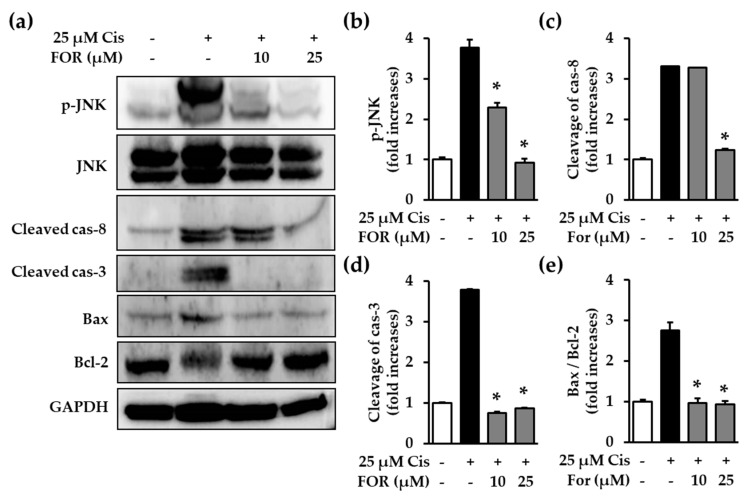
Formononetin inhibits caspase activation and Bcl-2 family-mediated apoptosis. Cells were treated with 25 μM cisplatin in the presence of formononetin for 24 h. (**a**) Immunoreactive bands were detected using Western blot analysis; (**b**–**e**) immunoreactive bands were quantitatively analyzed for c-Jun N-terminal kinase (JNK) phosphorylation (**b**) and cleavage of caspase-8 (**c**), caspase-3 (**d**), and the ratio of Bax to Bcl-2 protein expression (**e**) (mean ± S.E.M., * *p* < 0.05 compared with cisplatin-treated cells).

Although formononetin prevented cisplatin-mediated apoptosis in LLC-PK1 cells, the underlying molecular mechanism was unclear. There are several suggested mechanisms for cisplatin-mediated apoptosis, including increased phosphorylation of mitogen-activated protein kinases (MAPK) proteins, activation of caspase-8 and caspase-3, and regulation of the Bcl-2 family pathway [20,28]. Therefore, we performed a Western blot analysis to investigate the molecular mechanism of formononetin-mediated protection against cisplatin-induced apoptotic LLC-PK1 cell death. Cisplatin was reported to induce JNK activation and a specific inhibitor for JNK prevented apoptosis during cisplatin-induced kidney damage [29]. Our results showed that formononetin significantly reduced the JNK phosphorylation increased by cisplatin in LLC-PK1 cells (Figure 6a,b). Moreover, cleavage of caspase-8 and caspase-3 proteins was markedly increased in cisplatin-treated LLC-PK1 cells, whereas these effects of cisplatin were significantly reduced by formononetin (Figure 6a,c,d). Treatment with cisplatin also increased Bax protein expression and decreased Bcl-2 protein expression (Figure 6a). The increased ratio of Bax to Bcl-2 protein expression is an indicator of apoptotic cell death [30]. In this sense, the data revealed that formononetin diminished the ratio of Bax to Bcl-2, which was increased by cisplatin in LLC-PK1 cells (Figure 6e). These data indicate that formononetin prevents cisplatin-induced apoptotic cell death via inhibition of the JNK-mediated extrinsic caspase cascade as well as the Bcl-2 family pathway.

## 3. Materials and Methods

### 3.1. Chemicals and Reagents

Cisplatin, *N*-acetylcysteine (NAC), Hoechst 33342, and 2′,7′-dichlorofluorescin diacetate (DCFDA) were purchased from Sigma Aldrich (St Louis, MO, USA). Dulbecco’s modified Eagle medium (DMEM) and RPMI1640 medium were purchased from Cellgro (Manassas, VA, USA). Fetal bovine serum (FBS) and Tali apoptosis kit were purchased from Invitrogen Co. (Grand Island, NY, USA). RIPA buffer and antibodies for c-Jun-N-terminal kinase (JNK), phospho-JNK, cleaved caspase-8, cleaved caspase-3, Bcl-2, Bax, glyceraldehyde 3-phosphate dehydrogenase (GAPDH), and anti-rabbit secondary antibodies conjugated with horseradish peroxidase (HRP) were purchased from Cell Signaling Technology (Boston, MA, USA). The EZ-Cytox cell viability assay kit was purchased from Dail Lab Service Co. (Seoul, Korea). Enhanced chemiluminocence (ECL) Advance Western Blotting Detection Reagents were purchased from GE Healthcare (Cambridge, UK).

### 3.2. Cell Cultures

The LLC-PK1 cells (a pig kidney epithelium cell line) and HeLa cells (human cervical cancer cell line) were purchased from the American Type Culture Collection (ATCC; Manassas, VA, USA). Two other cervical cancer cells, SiHa and CaSKi cells, were obtained from the Korean Cell Line Bank (Seoul, Korea). Cells were grown in a DMEM (for LLC-PK1, HeLa, and SiHa cells) or RPMI1640 medium (for CaSKi cells) medium containing 10% FBS, 1% penicillin/streptomycin, and 4 mM l-glutamine. Cells were maintained at 37 °C in a humidified incubator under 5% CO_2_, 95% atmosphere.

### 3.3. Cell Viability Assay

Cells were plated onto 96-well plates and incubated for 24 h to adhere. Cells were exposed to 25 μM cisplatin for 24 h after the preincubation with the indicated concentrations of formononetin for 2 h. The cells were then incubated with 10 μL Ez-Cytox cell viability assay solution for an additional 30 min. The absorbance was obtained using an E-Max microplate reader (Molecular Devices, Sunnyvale, CA, USA) at a wavelength of 450 nm. Cell viability was represented by a percentage of control cells.

### 3.4. Determination of Intracellular Reactive Oxygen Species

The LLC-PK1 cells were seeded onto clear-bottomed 96-well black plates at a density of 1 × 10^4^ cells per well. After the exposure to 25 μM cisplatin for 24 h in the absence or presence of 10 and 25 μM formonetin, the cells were stained with 2.5 μM 2′,7′-dichlorofluorescin diacetate (H_2_DCFDA). A fluorescent intensity was measured at an excitation wavelength of 485 nm and an emission wavelength of 535 nm using SPARK 10 M fluorescence plate reader (Tecan, Männedorf, Switzerland) and represented by a fold-increase compared with control cells. Fluorescent images were obtained using an IX51 fluorescent microscope (Olympus, Tokyo, Japan) equipped with a CCD camera.

### 3.5. Nuclear Staining with Hoechst 33342

LLC-PK1 cells were plated onto 6-well plates at a density of 4 × 10^5^ cells per well and incubated for 24 h to adhere. The cells were exposed to 25 μM cisplatin for 24 h in the presence of 10 and 25 μM formononetin and then stained with Hoeschst 33342. Fluorescent images were obtained using a IX51 fluorescent microscope (Olympus) equipped with a CCD camera.

### 3.6. Quantification of Apoptotic Cell Death

The LLC-PK1 cells were seeded on 6-well plates at a density of 4 × 10^5^ cells per well and exposed to 25 μM cisplatin for 24 h in the absence or presence of 10 and 25 μM formononetin. Cells were then stained with annexin V-Alexa Fluor 488 and propidium iodide under dark condition. Apoptotic cells were analyzed using a Tali Image-Based Cytometer (Invitrogen, CA, USA) and represented by the percentage of annexin V-positive cells.

### 3.7. Western Blotting Analysis

The LLC-PK1 cells were plated onto 6-well plates at a density of 4 × 10^5^ cells per well. The cells were treated with 25 μM cisplatin for 24 h in the absence or presence of 10 and 25 μM formonetin. Cells were harvested and lysed using a RIPA buffer containing a freshly added protease inhibitor cocktail and 1 mM phenylmethylsulfonyl fluoride (PMSF). Equal amounts of proteins were separated by SDS-PAGE gel electrophoresis and further transferred onto a polyvinylidene difluoride membrane (Merck Millipore, Darmstadt, Germany). The membranes were blocked with tris-buffered saline containing 5% skim milk and subsequently incubated with primary antibodies against c-Jun N-terminal kinase (JNK), phospho-JNK, JNK, cleaved caspase-8, cleaved caspase-3, Bcl-2, Bax, and GAPDH. The membranes were then incubated with appropriated horseradish peroxidase-conjugated secondary antibodies. Immunoreactive bands were visualized using ECL Advance Western Blotting Detection Reagents and a FUSION Solo Chemiluminescence System (PEQLAB Biotechnologie GmbH, Erlangen, Germany) and quantitatively analyzed using ImageJ software (National Institutes of Health, Bethesda, MD, USA).

### 3.8. Statistical Analysis

The data are presented as the mean ± standard error mean (S.E.M.). Statistical significance was determined using the Student’s *t*-test. *p*-Values less than 0.05 were considered statistically significant.

## 4. Conclusions

In conclusion, this study demonstrated that formononetin is a potent protectant against cisplatin-induced cell death through inhibiting intracellular ROS accumulation in pig kidney epithelial LLC-PK1 cells. Furthermore, formononetin affects neither cell death nor the anticancer effect of cisplatin in human cervical cancer cell lines, including HeLa, SiHa, and CaSKi cells. Therefore, this study suggested that formononetin may be a beneficial regimen to ameliorate the anticancer effect of cisplatin in treating cervical cancer through the prevention of nephrotoxicity as an adverse effect of cisplatin.
